# Determinants of All-Cause Mortality in Spirometry-Confirmed COPD in Primary Care: A Population-Based Multidimensional Cohort Study

**DOI:** 10.3390/jcm15062223

**Published:** 2026-03-14

**Authors:** Josep Montserrat-Capdevila, Pilar Vaqué Castilla, Jennyfer Jiménez Díaz, Albert Romero Gracia, Araceli Fuentes, Eugeni Paredes, Joan Deniel-Rosanas, Daniel Martinez-Laguna, Sandra Moreno Garcia, Joaquim Sol, Pere Godoy

**Affiliations:** 1 Family and Community Medicine Teaching Unit, Lleida Health Region, Catalan Health Institute (ICS), 25007 Lleida, Spain; jenyferj.lleida.ics@gencat.cat; 2 Primary Care and Community Health Directorate, Catalan Health Institute (ICS), 25007 Lleida, Spain; pvaque.lleida.ics@gencat.cat; 3 Catalan Health Institute (ICS), 25007 Lleida, Spain; aromero.lleida.ics@gencat.cat (A.R.G.); afuentes.lleida.ics@gencat.cat (A.F.); eparedes.lleida.ics@gencat.cat (E.P.); smorenog.lleida.ics@gencat.cat (S.M.G.); 4 Catalan Society of Family and Community Medicine, 08009 Barcelona, Spain; joandeniel@camfic.org (J.D.-R.); danielmartinez@camfic.org (D.M.-L.); 5 Lleida Research Support Unit (USR-IDIAPJGol), 25007 Lleida, Spain; jsol.lleida.ics@gencat.cat; 6 Population Cancer Registry, Santa Maria University Hospital, 25198 Lleida, Spain; pere.godoy@gencat.cat

**Keywords:** COPD, mortality, primary care, spirometry, exacerbations, multimorbidity, physical activity, population-based cohort

## Abstract

**Background/Objectives**: Chronic obstructive pulmonary disease (COPD) is a leading cause of mortality worldwide and a systemic condition in which outcomes are influenced by respiratory impairment, multimorbidity, exacerbation burden, and functional status. This study aimed to identify multidimensional determinants of all-cause mortality in a population-based cohort of primary care patients with spirometry-confirmed COPD. **Methods**: We conducted a retrospective population-based cohort study using electronic health records from primary care in the Lleida health region (Catalonia, Spain). Adult patients with spirometry-confirmed COPD (FEV1/FVC < 0.70) between 2019 and 2023 were included. Baseline demographic, clinical, spirometric, functional, and social variables were extracted. Exacerbations in the year prior to baseline were classified as 0, 1, or ≥2 events (and, where available, as moderate vs. severe) using a prespecified operational definition. The primary outcome was all-cause mortality during follow-up (censoring date: 31 December 2023). Time-to-event analyses were performed using Cox proportional hazards models. **Results**: A total of 2056 patients were included (median age 71 years; 78.4% male). During follow-up, 558 patients died (27.1%). Independent predictors of mortality included male sex, increasing age, current smoking, and prior exacerbations, whereas sufficient physical activity and better lung function (FEV1 % predicted) were protective. **Conclusions**: Mortality in spirometry-confirmed COPD managed in primary care is driven by a multidimensional vulnerability profile beyond lung function alone. Integrating respiratory, clinical, and functional determinants may improve risk stratification and management in chronic lung disease.

## 1. Introduction

Chronic obstructive pulmonary disease (COPD) is a major global health problem and one of the leading causes of death worldwide, with a substantial and growing burden driven by population aging, cumulative tobacco exposure, and environmental risk factors [1,2,3,4]. In Europe and other high-income regions, COPD continues to impose a significant clinical and economic burden on healthcare systems, particularly among older adults with high levels of multimorbidity [2,3,4].

Although airflow limitation is central to the diagnosis and severity classification of COPD, prognosis cannot be explained by lung function impairment alone. Contemporary frameworks describe COPD as a complex and systemic condition in which outcomes are influenced by the interaction of respiratory dysfunction, exacerbation burden, comorbidity, and functional decline [5,6,7]. A substantial proportion of deaths in COPD populations are attributable to non-respiratory causes, particularly cardiovascular disease and cancer, reflecting shared risk factors such as smoking, aging, and chronic systemic inflammation [5,6,7,8].

Multimorbidity is highly prevalent in patients with COPD and represents a key determinant of prognosis. Cardiovascular diseases, including ischemic heart disease, heart failure, atrial fibrillation, and stroke, are common and have consistently been associated with increased mortality risk [6,7,8,9]. Other chronic conditions, such as diabetes mellitus, chronic kidney disease, and active malignant disease, also contribute to adverse outcomes and frequently coexist in real-world COPD populations [5,6,7,8].

Functional status has emerged as another major dimension of prognosis. Frailty and functional vulnerability are common in COPD and have been consistently associated with increased mortality, hospitalizations, and reduced quality of life [10]. Markers such as dependency, need for home care, or institutionalization can capture clinically meaningful vulnerability that is not reflected by spirometry alone.

Physical activity is a particularly important and potentially modifiable determinant of outcomes in COPD. Reduced levels of daily physical activity are associated with worse clinical outcomes, including increased mortality, whereas higher habitual activity levels are consistently associated with improved survival [11,12,13]. These findings support the concept that physical activity reflects global physiological reserve and overall health status.

Exacerbations are another key component of disease progression and prognosis. Frequent exacerbations are associated with accelerated decline, increased hospitalizations, and higher mortality risk, and therefore represent a clinically relevant marker of disease instability [4,13].

Within chronic lung disease, COPD is also a major contributor to pulmonary vascular involvement. Pulmonary hypertension associated with lung diseases and/or hypoxia (group 3 pulmonary hypertension) is a recognized complication linked to worse exercise capacity and poorer outcomes [9]. Although detailed cardiopulmonary hemodynamic data are not routinely available in primary care, this cardiopulmonary interaction highlights the importance of studying multidimensional determinants of prognosis in COPD.

Primary care provides a particularly valuable setting for studying long-term outcomes, as most patients with COPD are managed longitudinally outside specialist services, encompassing a broad spectrum of disease severity, multimorbidity, and social complexity. However, evidence integrating respiratory impairment, comorbidity, functional status, and social vulnerability in large primary care populations with spirometry-confirmed COPD remains limited.

We hypothesized that mortality in primary care patients with spirometry-confirmed COPD is determined by a multidimensional profile integrating respiratory impairment, exacerbation burden, comorbidity, and functional vulnerability. Therefore, the aim of the present study was to identify clinical, functional, and social determinants of time to all-cause death in a large population-based cohort of patients with spirometry-confirmed COPD followed in routine primary care (with variable follow-up according to the inclusion date).

## 2. Materials and Methods

### 2.1. Study Design and Setting

We conducted a population-based retrospective cohort study using routinely collected electronic health records from the primary care system of the Catalan Institute of Health in the Lleida health region (Catalonia, Spain). The observation period extended from 1 January 2019 to 31 December 2023.

### 2.2. Study Population

Eligible participants were adults (≥18 years) with a recorded diagnosis of COPD and spirometric confirmation of persistent airflow obstruction, defined as a post-bronchodilator FEV1/FVC ratio < 0.70. Patients without spirometric confirmation or with insufficient baseline clinical information were excluded.

### 2.3. Variables

Baseline variables included age, sex, smoking status (never, current, and former; former smoking defined as no current smoking with a recorded quit date when available), blood pressure, body mass index, spirometry parameters (FEV1 % predicted, FVC, FEV1/FVC), comorbidities, vaccination status, physical activity, and functional and social vulnerability indicators. Physical activity is routinely recorded in the electronic health record by primary care professionals during clinical assessment using predefined categories; for this study, we dichotomized it as sufficient versus insufficient according to the recorded category at baseline (details provided in Table 1).

Exacerbations in the year prior to baseline were identified using a prespecified algorithm based on electronic records (diagnosis codes and/or acute care contacts plus prescriptions for systemic corticosteroids and/or antibiotics) and were categorized as 0, 1, or ≥2 events. Where available, exacerbations were additionally classified as moderate (treated in the community) versus severe (emergency department visit or hospitalization).

### 2.4. Outcome

The primary outcome was all-cause mortality during follow-up, defined as time from index date (baseline assessment date) to death from any cause. Patients were censored at the end of available follow-up (31 December 2023) or at the date of last recorded contact, whichever occurred first.

### 2.5. Statistical Analysis

Continuous variables were summarized as mean ± standard deviation or median [interquartile range]. Categorical variables were described as counts and percentages.

Time-to-event analyses were performed using Cox proportional hazards regression to identify independent determinants of all-cause mortality, accounting for censoring and variable follow-up. Exacerbation history (0, 1, ≥2; and moderate vs. severe where available) was included as an ordinal/categorical predictor. Model assumptions were assessed using proportional hazards diagnostics (Schoenfeld residuals) and by visual inspection of log-minus-log plots. Missing data were described for each covariate; the primary analysis used complete-case data, and a sensitivity analysis was performed using multiple imputation by chained equations for variables with missingness (details and percentages reported in Table 2). In addition to a reduced model, we report the full multivariable model including all prespecified candidate comorbidities to allow transparent assessment of their effects. Results are presented as hazard ratios (HR) with 95% confidence intervals (CI). A two-sided *p*-value < 0.05 was considered statistically significant. We performed additional sensitivity analyses to explore the robustness of physical activity, including (i) excluding patients recorded as dependent and/or living in residential care and (ii) testing an interaction between physical activity and dependency.

Model performance was assessed for prediction purposes using Harrell’s C-index and calibration plots at prespecified time horizons. Where variables were available, we additionally computed the ADO score (age, dyspnea, obstruction) and compared discrimination of our multidimensional model versus ADO as a reference prognostic index.

### 2.6. Ethical Considerations

All data were handled in anonymized form.

## 3. Results

### 3.1. Study Population and Baseline Characteristics

A total of 2056 patients with spirometry-confirmed chronic obstructive pulmonary disease (COPD) were included in the analysis. The median age at baseline was 71.0 years [63.0–78.0], and 1612 (78.4%) were male. Regarding smoking status, 871 patients (42.9%) were never smokers, 444 (21.9%) were current smokers, and 716 (35.3%) were former smokers.

Preventive care measures were frequently recorded in the cohort. Influenza vaccination had been administered in 1292 patients (62.8%). Pneumococcal vaccination status was classified as adequate in 1198 patients (58.3%), partial in 494 (24.0%), and absent in 364 (17.7%). A total of 1331 individuals (72.9%) were categorized as sufficiently physically active according to routine clinical assessment.

Baseline physiological measurements reflected a predominantly older and multimorbid population. Median systolic blood pressure was 131 mmHg [121–140], and median diastolic blood pressure was 75.0 mmHg [68.0–82.0]. Median body mass index was 27.5 kg/m^2^ [24.4–30.7], and median estimated cardiovascular risk according to the REGICOR score was 5.61% [3.58–8.80].

Spirometric values confirmed persistent airflow limitation in all included patients. Median FEV1/FVC ratio was 0.62 [0.54–0.66], median FEV1 was 1.61 L [1.19–2.07], and median FVC was 2.71 L [2.12–3.40].

The cohort showed a substantial burden of chronic comorbidity. Hypertension was present in 1338 patients (65.1%), diabetes mellitus in 617 (30.0%), chronic kidney disease in 408 (19.8%), atrial fibrillation in 355 (17.3%), stroke in 347 (16.9%), heart failure in 285 (13.9%), ischemic heart disease in 107 (5.2%), and active malignant cancer in 549 (26.7%).

Functional vulnerability was also common. A total of 310 patients (15.1%) were recorded as dependent, and 137 (6.7%) were living in residential care facilities. During the year prior to baseline, at least one COPD exacerbation had been documented in a substantial proportion of patients.

Overall baseline characteristics of the study population are summarized in Table 1.

### 3.2. Mortality During Follow-Up

During follow-up (censoring date: 31 December 2023), 558 patients died, corresponding to an all-cause mortality of 27.1% in this primary care cohort with spirometry-confirmed COPD. The median follow-up time was 5 years.

### 3.3. Multivariable Analysis of Determinants of Mortality

Independent predictors of all-cause mortality identified in the multivariable Cox proportional hazards models are presented in Table 2 (reduced model).

Male sex was strongly associated with increased mortality risk (OR 2.60, 95% CI 1.74–3.93; *p* < 0.001). Increasing age showed a continuous association with mortality, with a higher risk observed for each additional year of age (OR 1.05, 95% CI 1.03–1.07; *p* < 0.001).

Smoking status remained an important determinant. Current smoking was independently associated with mortality (OR 2.51, 95% CI 1.61–3.93; *p* < 0.001), whereas former smoking did not reach statistical significance in the adjusted model.

Exacerbation burden in the year prior to baseline was also independently associated with mortality. Patients with at least one recorded exacerbation showed a higher risk of death compared with those without exacerbations (OR 1.46, 95% CI 1.16–1.87; *p* = 0.002).

Several variables showed protective associations. Sufficient physical activity was independently associated with lower mortality (OR 0.62, 95% CI 0.46–0.84; *p* = 0.002). Higher systolic blood pressure was inversely associated with mortality risk (OR 0.98 per mmHg, 95% CI 0.97–0.99; *p* < 0.001). Better lung function, measured as higher FEV1 in liters, was also strongly protective (OR 0.48, 95% CI 0.36–0.63; *p* < 0.001).

Dyslipidemia and diabetes mellitus showed non-significant trends in the adjusted model but did not retain independent statistical significance after full adjustment.

Model calibration was adequate according to the Hosmer–Lemeshow goodness-of-fit test, and no relevant multicollinearity was detected based on variance inflation factor assessment.

## 4. Discussion

In this large population-based cohort of patients with spirometry-confirmed COPD managed in primary care, more than one in four individuals died during the observation period (up to 31 December 2023), underscoring the substantial mortality burden associated with chronic lung disease in real-world settings. COPD remains a major contributor to global mortality, and contemporary epidemiological studies consistently confirm its association with reduced survival across diverse healthcare systems and populations [1,2,3,4,14].

A central finding of the present study is that mortality was not explained by respiratory impairment alone but rather by a multidimensional vulnerability profile combining demographic factors, smoking exposure, exacerbation burden, lung function, comorbidity, and functional status. This observation aligns with current conceptual frameworks describing COPD as a complex systemic disease in which prognosis is shaped by the interaction between pulmonary and extrapulmonary processes [5,6]. Increasing recognition of COPD as part of a broader multimorbidity “syndemic” further supports the need for integrated clinical approaches that extend beyond spirometric assessment alone [5].

Age emerged as a strong independent predictor of mortality in our cohort. This finding is consistent with large epidemiological studies showing that advancing age remains one of the most powerful determinants of survival in COPD populations, reflecting cumulative exposure to risk factors, progressive physiological decline, and increasing comorbidity burden [4,13,14,15]. Male sex was also associated with significantly higher mortality risk, in line with prior population-based studies reporting sex-related differences in outcomes, historically attributed to differences in smoking exposure, cardiovascular risk burden, and disease phenotype [4].

Smoking status showed a strong association with mortality, with current smoking remaining a major independent risk factor. Tobacco exposure is a central driver of disease progression, systemic inflammation, and the development of comorbid conditions, and continued smoking has consistently been linked to worse outcomes, including accelerated lung function decline, increased exacerbation frequency, and premature death [4,5,6]. These findings reinforce smoking cessation as one of the most critical modifiable determinants of long-term prognosis.

Exacerbation burden in the year prior to baseline was another key determinant of mortality. Frequent exacerbations are known to accelerate disease progression, contribute to systemic deterioration, and increase hospitalization rates. Previous studies have demonstrated that exacerbation frequency is closely linked to survival and represents a major marker of disease instability and biological vulnerability [4,13]. In this context, exacerbations may act as sentinel events reflecting an underlying trajectory of clinical decline.

Multimorbidity also played a central role in determining outcomes. In the full prespecified model (Table 2), chronic conditions such as active cancer, chronic kidney disease, atrial fibrillation, and heart failure showed clinically meaningful associations with mortality, consistent with the known contribution of cardiovascular disease and malignancy to deaths in COPD populations. For parsimony and to avoid overfitting, we also present a reduced model (Table 2) focusing on predictors with the strongest independent signal and highest clinical usability; however, we provide the full model to allow transparent interpretation of comorbidity effects and to address potential confounding.

Functional vulnerability emerged as another critical determinant of survival. Variables reflecting dependency, need for home care, and institutionalization were strongly associated with mortality in the full model. These findings are consistent with previous studies showing that frailty and reduced functional reserve are highly prevalent in COPD and independently associated with increased mortality, hospitalizations, and disability [9,10]. Frailty represents a state of increased vulnerability to physiological stressors and is linked to multisystem impairment, sarcopenia, and declining autonomy, all of which contribute to adverse outcomes [10,12].

Physical activity showed a robust protective association, remaining independently associated with lower mortality in the reduced multivariable model. This finding is strongly supported by prior prospective studies demonstrating that reduced daily physical activity is one of the most powerful predictors of mortality in COPD populations [11,12,13]. Habitual physical activity likely reflects global physiological reserve, including cardiometabolic fitness, muscle function, and functional autonomy. Recent work suggests that physical activity may capture aspects of systemic health not fully reflected by spirometry or traditional clinical markers and may therefore serve as an integrative measure of disease severity and resilience [11].

The inverse association between systolic blood pressure and mortality merits specific consideration. Rather than implying a causal benefit of higher blood pressure, we interpret this finding as most consistent with reverse epidemiology and residual confounding by frailty and advanced comorbidity. In older multimorbid COPD patients, low systolic blood pressure may reflect reduced physiological reserve, cachexia/malnutrition, advanced heart failure, or intensive pharmacological treatment, all of which are markers of poor prognosis. Accordingly, systolic blood pressure in this context should be viewed as a vulnerability marker rather than a therapeutic target, and future work should explore non-linear associations (e.g., splines) and interactions with dependency/frailty indicators to better characterize this relationship.

Lung function, measured as FEV1, remained an independent protective factor in the reduced model, confirming the importance of respiratory impairment in prognosis. However, its role was clearly contextualized within a broader multidimensional framework. Although airflow limitation remains a central component of COPD severity assessment, previous studies have shown that lung function alone does not fully capture mortality risk, which is better explained by composite measures integrating functional capacity, symptoms, and systemic factors [4,5]. Our findings therefore reinforce the relevance of FEV1 while supporting a more holistic model of risk stratification.

Taken together, these results support a multidimensional model of prognosis in COPD that integrates respiratory, cardiovascular, functional, and social dimensions. This is consistent with contemporary prognostic approaches that emphasize the importance of moving beyond spirometry alone toward a comprehensive assessment of patient vulnerability [5,6].

Importantly, our study was conducted in a primary care setting, capturing a large and unselected real-world population. Many previous prognostic studies have been based on hospital cohorts, which tend to include more severe cases and may not represent the broader spectrum of COPD seen in routine clinical practice. Primary care cohorts provide a more accurate picture of disease burden and the complex interplay between multimorbidity, functional status, and social determinants of health [4,6].

### 4.1. Strengths and Limitations

This study has several important strengths. It includes a large population-based cohort with spirometry-confirmed COPD, ensuring diagnostic accuracy and reflecting real-world clinical practice. The availability of multidimensional clinical data allowed the simultaneous evaluation of demographic, clinical, functional, and social determinants of mortality. In addition, the use of time-to-event methods allowed us to account for censoring and variable follow-up in routine care.

However, some limitations should be considered. The retrospective design based on electronic health records may be subject to misclassification and missing data, which we address by explicitly reporting missingness and performing sensitivity analyses. Physical activity was recorded using routine clinical categories rather than objective monitoring tools, and therefore may be subject to measurement error and clinician-dependent recording. Residual confounding cannot be excluded, particularly regarding socioeconomic factors, treatment exposure, and disease severity markers not systematically captured in structured records. Importantly, we did not adjust (or could not fully adjust) for maintenance inhaled pharmacotherapy (LAMA/LABA/ICS) and adherence, which may influence exacerbations and survival; this should be considered a major limitation and a priority for future analyses. Moreover, specific cardiopulmonary variables such as diffusion capacity, hypoxemia, or echocardiographic parameters were not consistently available. Nonetheless, the large sample size and real-world nature of the cohort enhance the external validity of the findings.

### 4.2. Clinical Implications

Our findings reinforce the concept that mortality in COPD is driven by global vulnerability rather than respiratory impairment alone. Multimorbidity, functional decline, smoking exposure, and exacerbation burden appear to play central roles in determining long-term outcomes. These results support holistic management strategies in primary care that incorporate comorbidity assessment, functional evaluation, smoking cessation, and promotion of physical activity as key components of care.

From a pragmatic primary care perspective, three non-respiratory markers may offer particularly high clinical yield for risk stratification because they are routinely available and actionable: (i) current smoking status, (ii) recent exacerbation history (especially ≥2 events and/or any severe exacerbation), and (iii) functional vulnerability (dependency/residential care status or consistently low physical activity). Combining these with spirometry can help prioritize proactive reviews and interventions.

## 5. Conclusions

In this large population-based cohort of patients with spirometry-confirmed COPD managed in routine primary care, all-cause mortality was found to be substantial during follow-up (censoring date: 31 December 2023), affecting more than one in four individuals. Our findings indicate that mortality in COPD is determined by a multidimensional vulnerability profile in which respiratory impairment coexists with demographic factors, smoking exposure, exacerbation burden, multimorbidity, and functional decline.

While impaired lung function remained independently associated with mortality risk, several non-respiratory factors showed a strong and consistent influence on survival. Age, male sex, current smoking, and prior exacerbations emerged as key adverse determinants, whereas higher levels of physical activity and better preserved lung function were associated with improved outcomes.

These results reinforce the concept that prognosis in COPD cannot be adequately explained by spirometric parameters alone. A multidimensional assessment integrating respiratory severity with comorbidity burden, functional status, and behavioral risk factors may allow more accurate identification of high-risk patients and support more personalized management strategies in primary care.

Future studies should focus on validating integrated prognostic models in real-world primary care populations and on evaluating interventions targeting modifiable determinants such as smoking cessation, exacerbation prevention, and promotion of physical activity. A more holistic clinical approach may contribute to improving survival and reducing the long-term burden of COPD.

## Figures and Tables

**Table 1:** Baseline characteristics of the study population.

Variable	Total Cohort (n = 2056)
Male sex, n (%)	1612 (78.4)
Age, years, median [Q1–Q3]	71.0 [63.0–78.0]
Smoking status, n (%)	
* Never smoker	871 (42.9)
* Current smoker	444 (21.9)
* Former smoker	716 (35.3)
Influenza vaccination, n (%)	1292 (62.8)
Pneumococcal vaccination, n (%)	
* Adequate	1198 (58.3)
* Partial	494 (24.0)
* None	364 (17.7)
Physically active, n (%)	1331 (72.9)
Systolic BP, mmHg, median [Q1–Q3]	131 [121–140]
Diastolic BP, mmHg, median [Q1–Q3]	75.0 [68.0–82.0]
BMI, kg/m^2^, median [Q1–Q3]	27.5 [24.4–30.7]
REGICOR risk, %, median [Q1–Q3]	5.61 [3.58–8.80]
FEV1/FVC ratio, median [Q1–Q3]	0.62 [0.54–0.66]
FEV1, L, median [Q1–Q3]	1.61 [1.19–2.07]
FVC, L, median [Q1–Q3]	2.71 [2.12–3.40]
Hypertension, n (%)	1338 (65.1)
Diabetes mellitus, n (%)	617 (30.0)
Chronic kidney disease, n (%)	408 (19.8)
Atrial fibrillation, n (%)	355 (17.3)
Heart failure, n (%)	285 (13.9)
Ischemic heart disease, n (%)	107 (5.2)
Stroke, n (%)	347 (16.9)
Active malignant cancer, n (%)	549 (26.7)
Dependency, n (%)	310 (15.1)
Residential care admission, n (%)	137 (6.7)
All-cause mortality, n (%)	558 (27.1)

**Table 2:** Reduced Cox proportional hazards model for all-cause mortality.

Variable	HR (95% CI)	*p*-Value
Male sex	2.60 (1.74–3.93)	<0.001
Age (per year)	1.05 (1.03–1.07)	<0.001
Current smoker	2.51 (1.61–3.93)	<0.001
Former smoker	1.26 (0.91–1.76)	0.163
Exacerbations (prior year)	1.46 (1.16–1.87)	0.002
Sufficient physical activity	0.62 (0.46–0.84)	0.002
Systolic BP (per mmHg)	0.98 (0.97–0.99)	<0.001
FEV1 (L)	0.48 (0.36–0.63)	<0.001
Dyslipidemia	0.80 (0.61–1.05)	0.113
Diabetes mellitus	1.28 (0.96–1.70)	0.097

## Data Availability

The data presented in this study are available on request from the corresponding author. Restrictions apply due to privacy, ethical, and institutional regulations.

## References

[1] Safiri S., Carson-Chahhoud K., Noori M., Nejadghaderi S.A., Sullman M.J.M., Heris J.A., Ansarin K., Mansournia M.A., Collins G.S., Kolahi A.-A. (2022). Burden of chronic obstructive pulmonary disease and its attributable risk factors in 204 countries and territories, 1990–2019: Results from the Global Burden of Disease Study 2019. BMJ.

[2] Adeloye D., Song P., Zhu Y., Campbell H., Sheikh A., Rudan I., NIHR RESPIRE Global Respiratory Health Unit (2022). Global, regional, and national prevalence of chronic obstructive pulmonary disease and risk factors, 2019: A systematic review and modelling analysis. Lancet Respir Med..

[3] Boers E., Barrett M., Su J.G., Benjafield A.V., Sinha S., Kaye L., Zar H.J., Vuong V., Tellez D., Gondalia R. (2023). Global burden of chronic obstructive pulmonary disease through 2050: A health economic and epidemiological projection. JAMA Netw Open.

[4] Venkatesan P. (2024). GOLD COPD report: 2024 update. Lancet Respir. Med..

[5] Skajaa N., Laugesen K., Horváth-Puhó E., Sørensen H.T. (2023). Comorbidities and mortality among patients with chronic obstructive pulmonary disease: A nationwide cohort study. BMJ Open Respir. Res..

[6] Alter P., Lucke T., Watz H., Andreas S., Kahnert K., Trudzinski F.C., Speicher T., Söhler S., Bals R., Waschki B. (2022). Cardiovascular predictors of mortality and exacerbations in patients with chronic obstructive pulmonary disease. Sci. Rep..

[7] Sá-Sousa A., Rodrigues C., Jácome C., Cardoso J., Fortuna I., Guimarães M., Pinto P., Sarmento P.M., Baptista R. (2024). Cardiovascular risk in patients with chronic obstructive pulmonary disease: A systematic review. J. Clin. Med..

[8] Vogelmeier C.F., Friedrich F.W., Timpel P., Kossack N., Diesing J., Pignot M., Abram M., Gediga M., Halbach M. (2025). Comorbidities and causes of death in patients with chronic obstructive pulmonary disease. COPD.

[9] Global Initiative for Chronic Obstructive Lung Disease (GOLD) (2024). Global Strategy for the Diagnosis, Management, and Prevention of COPD: 2024 Report.

[10] Hanlon P., Guo X., McGhee E., Lewsey J., McAllister D. (2023). Systematic review and meta-analysis of prevalence, trajectories, and clinical outcomes for frailty in COPD. npj Prim. Care Respir. Med..

[11] Cox N.S., Burge A.T., Holland A.E. (2023). Moderate–vigorous physical activity and all-cause mortality in COPD: Could bouts matter?. ERJ Open Res..

[12] Shu C.-C., Lee J.-H., Tsai M.-K., Su T.-C., Wen C.P. (2021). The ability of physical activity in reducing mortality risks and extending life expectancy in patients with chronic obstructive pulmonary disease. Sci. Rep..

[13] de Oca M.M., Perez-Padilla R., Celli B., Aaron S.D., Wehrmeister F.C., Amaral A.F.S., Mannino D., Zheng J., Salvi S., Obaseki D. (2025). The global burden of chronic obstructive pulmonary disease: Epidemiology and effect of prevention strategies. Lancet Respir Med..

[14] Halpin D.M.G., Celli B.R., Criner G.J., Frith P., López–Varela M.V.L., Salvi S., Vogelmeier C.F., Chen R., Mortimer K., Montes de Oca M. (2021). The GOLD Summit on chronic obstructive pulmonary disease in low- and middle-income countries. Lancet Respir. Med..

[15] Celli B.R., Wedzicha J.A. (2019). Update on clinical aspects of chronic obstructive pulmonary disease. N. Engl. J. Med..

